# Metastatic potential of small posterior uveal melanomas

**DOI:** 10.1111/aos.17582

**Published:** 2025-08-21

**Authors:** Gustav Stålhammar, Helen Kalirai, Sarah E. Coupland, Salvatore Grisanti, Svenja Rebecca Sonntag, Ayseguel Tura, Antonio Eleuteri, Rumana N. Hussain, Heinrich Heimann, Hannah Coudé Adam

**Affiliations:** ^1^ Division of Eye and Vision, Department of Clinical Neuroscience Karolinska Institutet Stockholm Sweden; ^2^ Ocular Oncology Service, St. Erik Eye Hospital Stockholm Sweden; ^3^ St. Erik Ophthalmic Pathology Laboratory, St. Erik Eye Hospital Stockholm Sweden; ^4^ Liverpool Ocular Oncology Research Group, Department of Eye and Vision Science, University of Liverpool Liverpool UK; ^5^ Liverpool Clinical Laboratories, NHS University Hospitals of Liverpool Group Liverpool UK; ^6^ Department of Ophthalmology University Medical Center Schleswig‐Holstein Luebeck Germany; ^7^ Research and Innovation Department, NHS University Hospitals of Liverpool Group Liverpool UK; ^8^ Liverpool Ocular Oncology Centre, NHS University Hospitals of Liverpool Group Liverpool UK

**Keywords:** choroidal melanoma, metastases, monosomy 3, prognosis, size, uveal melanoma

## Abstract

**Purpose:**

To investigate the prevalence of aggressive traits in small posterior uveal melanomas (UM).

**Methods:**

This retrospective, multicentre cohort study included 804 patients with small posterior UM (≤9 mm in largest basal diameter, ≤3 mm in thickness) from centres in the UK, Germany, and Sweden. Chromosomal aberrations, cytomorphology, nuclear BAP1 expression and circulating tumour cells (CTC) were analysed.

**Results:**

Markers of poor prognosis, including monosomy 3, 8q gain, loss of BAP1 expression, and the presence of CTC, were detected in tumours as small as 3–4 mm in diameter and 1–1.5 mm in thickness. In the cytogenetically analysed subset, monosomy 3 was observed in 23% (41/175), 8q gain in 32% (23/72) and 6q aberrations in 13% (9/71); loss of nuclear BAP1 was seen in 22% (19/88); and CTC were detected in 50% (22/44). There was a strong association between tumour size and the number of clinical risk factors for lesion growth, such as subretinal fluid, orange pigment and acoustic hollowness on ultrasound, whereas these clinical features did not correlate with markers for poor prognosis. Monosomy of chromosome 3, gain of 8q, loss of 1p, gain or loss of 6q, gain or loss of 8p, loss of BAP1 expression, and cytomorphology were associated with worse survival.

**Conclusion:**

Aggressive traits are common in small UM, including those <10 mm^3^ in volume. Clinical risk factors traditionally used for assessing nevus growth appear more closely related to lesion size than metastatic potential. These findings underscore the importance of identifying novel clinical markers for metastatic risk.

## 1 INTRODUCTION

Uveal melanoma (UM) is the most common primary intraocular malignancy in adults, with the choroid being the most commonly affected site (McLaughlin et al., 2005; Singh et al., 2011). Distinguishing small choroidal melanomas from nevi can be challenging in the clinical setting, sometimes requiring a period of observation before the diagnosis can be established. Risk factors for growth include larger diameter and thickness, dome shape, presence of subretinal fluid, visual symptoms, orange pigment and low internal reflectivity on ultrasonography (US) (Al Harby et al., 2021; Roelofs et al., 2020; Stalhammar, 2021). This has led to the development of mnemonics for clinicians to remember important factors. One such mnemonic is TFSOM‐DIM, which stands for thickness >2 mm on US, subretinal fluid on optical coherence tomography (OCT), symptoms of vision loss, orange pigment on autofluorescence, melanoma hollow on US, and DIaMeter >5 mm by photography (Shields et al., 2019).

Despite successful local control of the ocular tumour, about half of all patients develop fatal metastases because of early dissemination of micrometastases, predominantly to the liver (Stalhammar, 2023; Uner et al., 2021). The likelihood of metastasis post‐treatment is strongly correlated with tumour size: 15‐year disease‐specific survival rates are about 85% for small tumours as defined by the American Joint Committee on Cancer (AJCC) T‐category and drop to 30% for the largest tumours (Kujala et al., 2013). The Cancer Genome Atlas (TCGA) study classifies UM into four prognostic groups with a 5‐year Kaplan–Meier rate of metastasis ranging from 4% to 60% (Shields et al., 2021).

Specific chromosomal alterations such as monosomy of chromosome 3 (M3), gains of 8q and 6p, along with the loss of 1p, strongly impact prognosis (Damato et al., 2007; Kilic et al., 2006; Prescher et al., 1996). Mutations in the *BAP1* gene located on chromosome 3, if occurring somatically in the remaining allele, markedly increase metastatic risk (Horsman et al., 1990; Jager et al., 2020). In contrast, *SF3B1* mutations are associated with a later onset of metastases, while an *EIF1AX* mutation in primary UM generally predicts a favourable prognosis and typically excludes simultaneous mutations in *SF3B1* and *BAP1* (Yavuzyigitoglu et al., 2016). Integration of clinical, histopathological, and genetic features of UM into a multiparametric algorithm enables individualized prognostication of patients and liver surveillance stratification with high sensitivity and specificity (Eleuteri et al., 2018; Robinson et al., 2023).

A multicentre study of 45 patients with small yet fatal choroidal melanomas (≤3 mm in thickness and ≤9 mm in diameter) suggested that tumours smaller than 3 mm in diameter are highly unlikely to metastasize, as all tumours included in the study were at least 3 mm in diameter at the time of treatment (Jouhi et al., 2019). However, because this study did not include non‐fatal cases, it remains uncertain whether this size truly represents the smallest threshold for metastatic potential or if smaller tumours are simply very rarely diagnosed. Subsequent estimates propose that somatic *BAP1* mutations can occur in tumours with volumes less than 6 mm^3^, approximately corresponding to a diameter of 3 mm and a thickness of 1.5 mm (Uner et al., 2021).

Building on this, the present study examines the prevalence of aggressive cytogenetic, immunohistochemical and cytomorphological traits, as well as circulating tumour cells (CTC), in small UM and investigates their relationship to metastatic mortality.

## 2 METHODS

### 2.1 Patients and tumours

Data on consecutively registered patients with small choroidal or ciliary body melanoma were collected from the Liverpool Ocular Oncology Centre, Liverpool, United Kingdom (*n* = 417), St. Erik Eye Hospital, Stockholm, Sweden (*n* = 343) and the University Medical Center Schleswig‐Holstein Lübeck, Germany (*n* = 44). Small tumours were defined as those with a thickness of ≤3 mm and an LBD of ≤9 mm, as described in the Small Fatal Choroidal Melanoma Study (Jouhi et al., 2019). Using our previously published equation, a tumour with an LBD of 9 mm and a thickness of 3 mm would have a volume of approximately 108 mm^3^ (Stalhammar et al., 2023). Patients with iris melanoma were not considered, whereas cases with melanomas believed to originate in the choroid or ciliary body but with secondary infiltration into the iris were included. The tumour origin was determined based on the geometric centre assessed by B‐scan ultrasound biomicroscopy (UBM), wide‐field retinal imaging and/or slit‐lamp biomicroscopy. The data included patient age and sex, LBD, tumour thickness, ciliary body involvement (CBI), extraocular extension (EXE, ≤5 mm, or >5 mm), follow‐up duration and clinical status (alive, dead from metastatic UM or dead from other causes).

All lesions were diagnosed as uveal melanomas (i.e. not as nevi) by specialized ocular oncologists using multimodal examinations (including B‐scan ultrasonography, OCT, standard‐ or wide‐field imaging, slit‐lamp biomicroscopy and/or UBM). In cases of diagnostic uncertainty, lesions were monitored for growth, with a melanoma diagnosis established upon documented enlargement. Alternatively, tissue for genetic and cytogenetic analyses was obtained by biopsy using transscleral or transvitreal approaches.

In the Liverpool cohort, chromosomal status was determined using multiplex ligation‐dependent probe amplification (MLPA) or with microsatellite analysis (MSA), as described previously (Thornton, Coupland, Heimann et al., 2020; Thornton, Coupland, Olohan et al., 2020). TCGA groups were assigned based on chromosome 3 and 8q status, as described previously (Bansal et al., 2024; Gelmi et al., 2022).

For the Stockholm cohort, immunohistochemical evaluation of BAP1 expression (classified as low if <33% of tumour cell nuclei stained versus high if ≥33% stained) and cytomorphological assessment (categorized as spindle, epithelioid or mixed) were performed in routine clinical practice, as previously described (See et al., 2020). Patients in the Stockholm cohort had been asked to self‐assess the duration of their visual symptoms, if any, on a scale of months at the time of melanoma diagnosis. A previous publication indicated that within this cohort, patients reporting a shadow in the visual field as the presenting symptom had worse survival, possibly reflecting the presence of retinal detachment—which in turn may be associated with increased microvascular density and vasculogenic mimicry (Fili et al., 2020; Sabazade, Gill et al., 2021; Sabazade, Herrspiegel et al., 2021).

For the Lübeck cohort, approximately 50 mL of peripheral blood was collected, and CTC were captured using dual‐immunomagnetic enrichment. A previous publication has reported results from this cohort (Grisanti et al., 2023). In this study, we correlate the presence of CTC with tumour volume, using data on LBD and thickness on an individual patient level. A flowchart of included patients and tumours, along with a summary of the data available from each cohort, is provided in Figure S1, with heatmaps in Table S1a (Liverpool cohort) and Table S1b (Lübeck cohort); a dedicated heatmap for the Stockholm cohort is provided in the Results section below.

This study was approved by ethical review authorities in each country (Stockholm: reference 20220672502 and 20230753702; Lübeck: 10200 and 13 219; Liverpool: 21/NW/0139 and 15/SC/0611) and followed the tenets of the Declaration of Helsinki. The Strengthening the Reporting of Observational Studies in Epidemiology (STROBE) guidelines were followed (checklist provided as a Data S1).

### 2.2 Assessment of tumour size

Tumour dimensions, including LBD, tumour thickness, ciliary body involvement (CBI) and EXE were assessed using the best available estimates at the time of diagnosis. All participating institutions predominantly employed B‐scan US for the measurement of LBD and tumour thickness. In case of exceptionally small tumours, OCT was utilised to provide a more accurate assessment of tumour thickness. Wide‐field retinal imaging, equipped with digital measurement tools, was further used for verifying tumour diameter and monitoring growth. In cases of anteriorly located tumours, UBM could be employed to assess potential spread into the ciliary body, iris, and anterior chamber angle. Similarly, magnetic resonance imaging (MRI) could be considered for evaluating suspected cases of EXE.

Tumour volumes were estimated with LBD and thickness (*t*), according to a previously used method (Stalhammar et al., 2023):

$$\text{Estimated volume of tumor}=\frac{\pi }{6}\times t\times \mathrm{LBD}\times \left(\mathrm{LBD}\times 0.85\right)$$

### 2.3 Follow‐up

Patient status had been contemporaneously registered with data provided by cause‐of‐death registries. The Swedish National Cause of Death Registry is continuously updated, with a maximum delay of three weeks from the time of death to record finalization. Thereby, the registry will be up to date about a patient's vital status, irrespective of clinical visits. The UK's registry, with monthly updates from the National Health Service (NHS) Cancer Registries, aligns with this approach.

### 2.4 Statistical analyses

All *p* values were two‐sided, with Holm–Bonferroni correction applied to adjust for multiple comparisons. Additionally, the significance level of 0.05 was Bonferroni‐corrected based on the total number of outcomes analysed within each cohort. For the Liverpool cohort, where eight comparisons were made, the corrected significance level was 0.00625 (0.05/8). In the Lübeck and Stockholm cohorts, five and four comparisons were conducted, resulting in corrected significance levels of 0.01 and 0.0125, respectively. Pearson's chi‐square (χ^2^) test for trend was used to evaluate the association between ordered groups (e.g. tumour size categories) and ordinal outcome variables (e.g. prognostic groups). The Cochran–Armitage test for trend was applied for binary outcomes across multiple ordered groups. Cumulative incidences of death from metastatic UM (MUM) and other causes were calculated from competing risk data with the crr package for R (R Foundation for Statistical Computing) (Scrucca et al., 2007). To visualize the relationships between variables, a heatmap and correlation matrix of Pearson correlation coefficients were generated using the reshape2 and ggplot2 packages in R.

## 3 RESULTS

### 3.1 Descriptive statistics

Of the 804 included UM patients, 414 (51%) were female. According to the inclusion criteria of LBD ≤9 mm and thickness ≤3 mm (corresponding to a volume of approximately 108 mm^3^), all tumours were classified as AJCC T1.

Among 782 patients with available survival data, 54 succumbed to metastatic disease, 96 to other causes, and 14 to unknown causes. At the time of data collection, 618 patients were still alive (Table 1). The median survival calculated among these 618 survivors was 3.9 years (interquartile range [IQR] 5.6); however, because this approach does not account for censoring, it does not accurately represent the true duration of follow‐up. When estimating follow‐up using the reverse Kaplan–Meier method, the median follow‐up times were 2.9 years (95% CI, 2.4–3.4) for the Liverpool cohort, 5.8 years (95% CI, 5.0–7.3) for the Lübeck cohort, and 6.8 years (95% CI, 5.6–7.9) for the Stockholm cohort. For survival analyses, the 14 patients who died from unknown causes were considered to have died from causes other than metastatic UM.

**TABLE 1:** Characteristics of included patients and tumours.

Cohort	Liverpool; *n* = 417	Stockholm; *n* = 343	Lübeck; *n* = 44
Sex, *n* (%)			
Female	210 (50)	173 (50)	31 (70)
Male	207 (50)	170 (50)	13 (30)
Age at diagnosis, mean (SD)	61 (14)	63 (13)	68 (12)
Tumour LBD, mean mm (SD, min, max)	7.0 (1.6, 1.2, 9.0)	6.9 (1.6, 2.0, 9.0)	5.4 (1.8, 2.0, 8.5)
Tumour thickness, mean mm (SD, min, max)	1.8 (0.6, 0.5, 3.0)	2.4 (0.5, 0.9. 3.0)	1.1 (0.7, 0.2, 2.5)
Tumour volume, mean mm^3^ (SD)	43 (25)	54 (26)	18 (18)
AJCC T‐category, *n* (%)			
T1a	383 (92)	320 (93)	44 (100)
T1b	30 (7)	14 (4)	0 (0)
T1c	2 (<1)	9 (3)	0 (0)
T1d	2 (<1)	0 (0)	0 (0)
T2a or higher	0 (0)	0 (0)	0 (0)
AJCC stage, *n* (%)			0 (0)
I	383 (92)	320 (93)	44 (100)
IIA	34 (8)	23 (7)	0 (0)
IIB or higher	0 (0)	0 (0)	0 (0)
Number of events			
Metastatic death	12	42	–
Other death	12	84	–
Death, unknown cause	14	0	–
Alive	379	217	22
N/a			22

Abbreviations: AJCC, American Joint Committee on Cancer; LBD, largest basal tumour diameter; SD, standard deviation.

From Liverpool, primary tumour cytogenetic data were available for 58 to 75 patients for chromosomes 1p, 6p, 6q, 8p and 8q, depending on the marker, and for 175 patients for chromosome 3. Complete data on both chromosome 3 and 8q—the two markers required for TCGA group classification—were available for 60 patients.

From Stockholm, cytomorphology and the immunohistochemical expression of BAP1 in tumour cell nuclei were recorded for 94 and 88 patients who underwent primary enucleation, respectively. For 62 of the primarily enucleated tumours—typically those with extensive growth around the optic disc rendering the lesion inaccessible to brachytherapy—clinical TFSOM‐DIM risk factors for nevus growth were determined based on fundus photos, wide‐field images and examinations using US and OCT. These factors for the growth of nevi were evaluated even though the lesions were histopathologically confirmed as melanomas (Figure S2). Further details on cytogenetic factors, cytomorphology and BAP1 expression are provided in Table 2.

**TABLE 2:** Distribution of cytomorphology, nBAP1‐expression, and chromosome status.

Cytomorphology, *n* = 94, *n* (%)	
Spindle	35 (37)
Mixed	34 (36)
Epithelioid	25 (27)
nBAP‐1 expression, *n* = 88, *n* (%)	
Retained	69 (78)
Loss	19 (22)
Chromosome 1p, *n* = 69, *n* (%)	
Normal/euploidy	57 (83)
Loss	9 (13)
Gain	3 (4)
Chromosome 3, *n* = 175, *n* (%)	
Normal/euploidy	134 (77)
Loss	41 (23)
Chromosome 6p, *n* = 58, *n* (%)	
Normal/euploidy	45 (78)
Loss	1 (2)
Gain	12 (21)
Chromosome 6q, *n* = 71, *n* (%)	
Normal/euploidy	62 (87)
Loss	5 (7)
Gain	4 (6)
Chromosome 8p, *n* = 75, *n* (%)	
Normal/euploidy	63 (84)
Loss	6 (8)
Gain	6 (8)
Chromosome 8q, *n* = 72, *n* (%)	
Normal/euploidy	47 (65)
Loss	2 (3)
Gain	23 (32)

*Note*: Cytomorphology and nBAP‐1 expression data are from the Stockholm cohort; chromosomal data are from the Liverpool cohort.

From Lübeck, complete data on the number of TFSOM‐DIM risk factors in the primary tumour, the presence or absence of CTC, and, when present, the chromosome 3 status of those CTC were available for all 44 included patients.

### 3.2 Smallest tumour size with aggressive traits

In the Liverpool cohort, monosomy 3 was identified in a UM as small as 3.3 mm in LBD and 1.4 mm in thickness, corresponding to an estimated volume of 7 mm^3^. This same tumour also exhibited gain of chromosome 8q and loss of 6q, and epithelioid cells, but no closed extracellular matrix loops were found on histopathological examination. Two years following local resection, the patient, a male aged over 70 years, remained alive. The smallest tumour that led to metastatic death was 4.3 mm in LBD and 1.0 mm in thickness (volume 8 mm^3^), diagnosed in a 69‐year‐old female who died 4.2 years post‐diagnosis; the chromosomal status of this tumour remains undetermined. The smallest tumours with aberrations of 1p, 6p and 8p had volumes of 10, 29, and 8 mm^3^, respectively.

**FIGURE 1:**
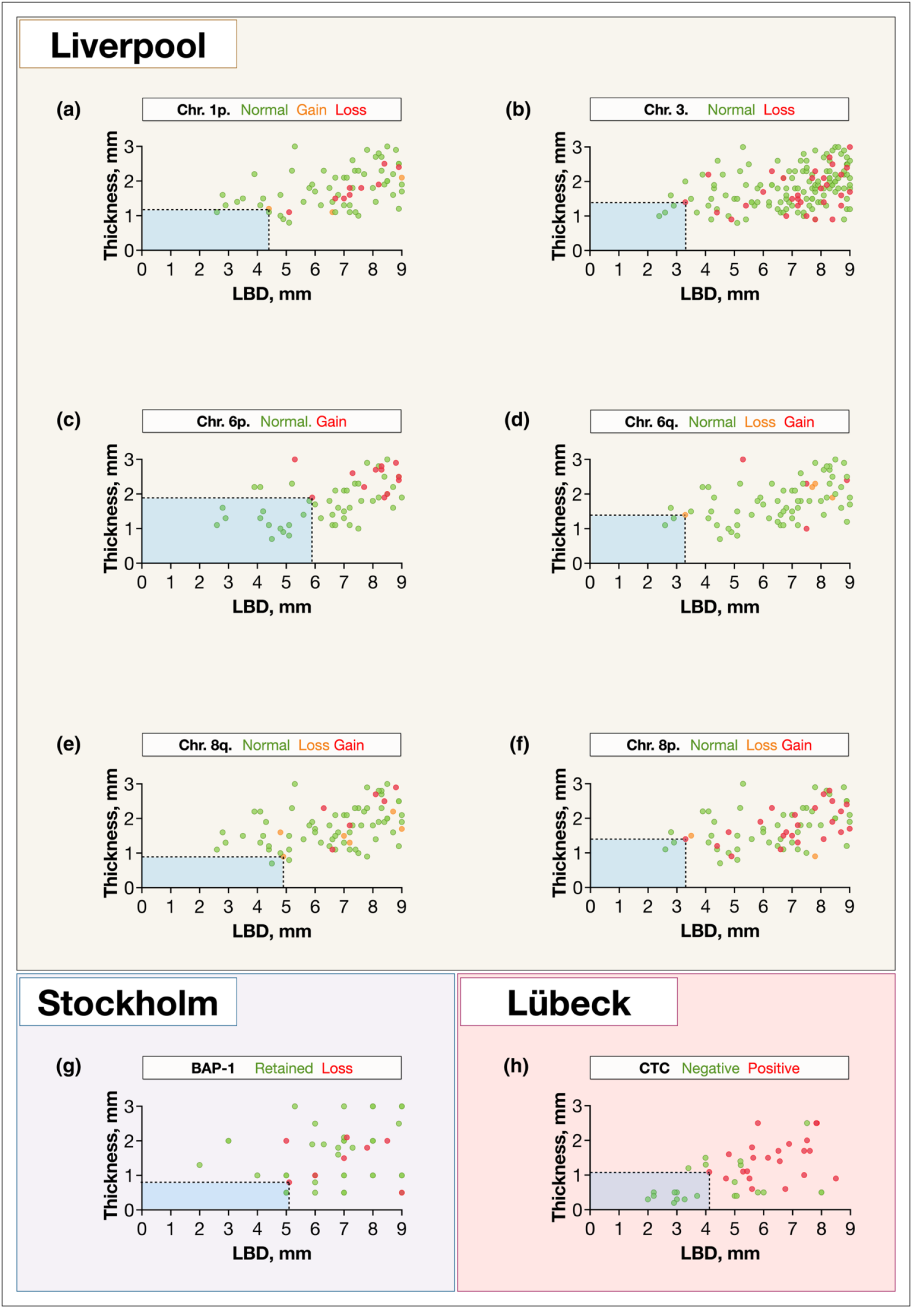
Distribution of chromosomal aberrations, immunohistochemical BAP1 expression and presence of circulating tumour cells (CTC) in relation to tumour size at diagnosis. (a) Chromosome (Chr.) 1p. (b) Chr. 3. (c) Chr. 6p. (d) Chr. 6q. (e) Chr. 8q. (f) Chr. 8p. (e) Immunohistochemical BAP1 expression. (f) CTC. Blue fields indicate tumour sizes with no observed chromosomal aberration, loss of BAP1 expression, or CTC, respectively. Note that these intervals contain a limited number of tumours, with the possible exception of those classified under chromosome 6p status and CTC.

In the Stockholm cohort, loss of nuclear BAP1 expression was identified in a tumour as small as 5.1 mm in LBD and 0.8 mm in thickness (volume 9 mm^3^). In the Lübeck cohort, the smallest tumour associated with CTC was 4.1 mm in LBD and 1.1 mm in thickness (volume 8 mm^3^, Figure 1).

### 3.3 Cumulative incidence of metastatic death by trait and symptom duration

**FIGURE 2:**
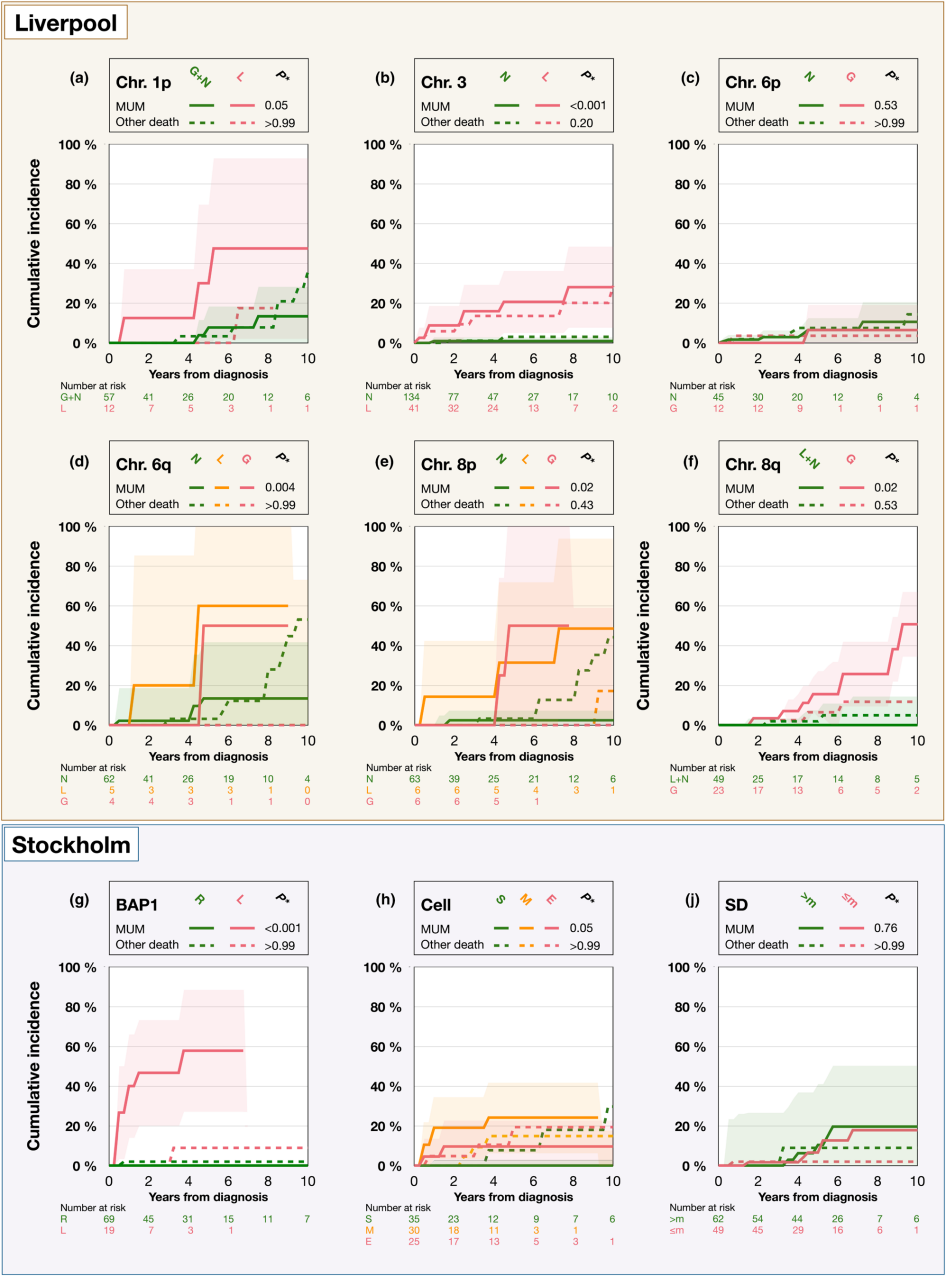
Competing risk cumulative incidence curves. Cumulative incidence of death from metastatic uveal melanoma (MUM) and any other cause (other death). (a) Chromosome (Chr.) 1p loss (L) versus gain or normal (G + N). (b) Chr. 3 loss versus normal. Chr. 6p gain versus normal (one patient with 6p loss was excluded due to small subgroup size). (d) Chr. 6q gain versus loss or normal (when combining 6q loss or gain into one category versus normal/, *p* = 0.005). (e) Chr. 8p gain versus loss or normal. (f) Chr. 8q gain versus normal. (g) Loss of immunohistochemical BAP1 expression in tumour cell nuclei versus retained expression (R). (h) Cytomorphological cell type, spindle‐like (S), versus mixed (M) or epithelioid (E). (j) Symptom duration (SD) ≤2 months versus >2 months. The duration of 2 months is the median value for 111 patients with available data. *Gray's test, Holm–Bonferroni corrected *p* value (6 comparisons in Liverpool cohort, 3 in Stockholm cohort). Coloured shadows indicate 95% confidence intervals.

In competing risk analyses, a higher cumulative incidence of metastatic death was observed in patients with tumours exhibiting monosomy 3 and alterations in chromosome 6q (either loss or gain). Additionally, tumours with loss of BAP1 expression were associated with a significantly higher cumulative incidence of metastatic death. No significant differences in the cumulative incidence of metastatic death were found in relation to chromosome 1p, 6p, 8p or 8q status, nor in patients who self‐reported a symptom duration of ≤2 months compared to >2 months (with 2 months being the median duration, Figure 2).

### 3.4 TCGA group and CTC

**FIGURE 3:**
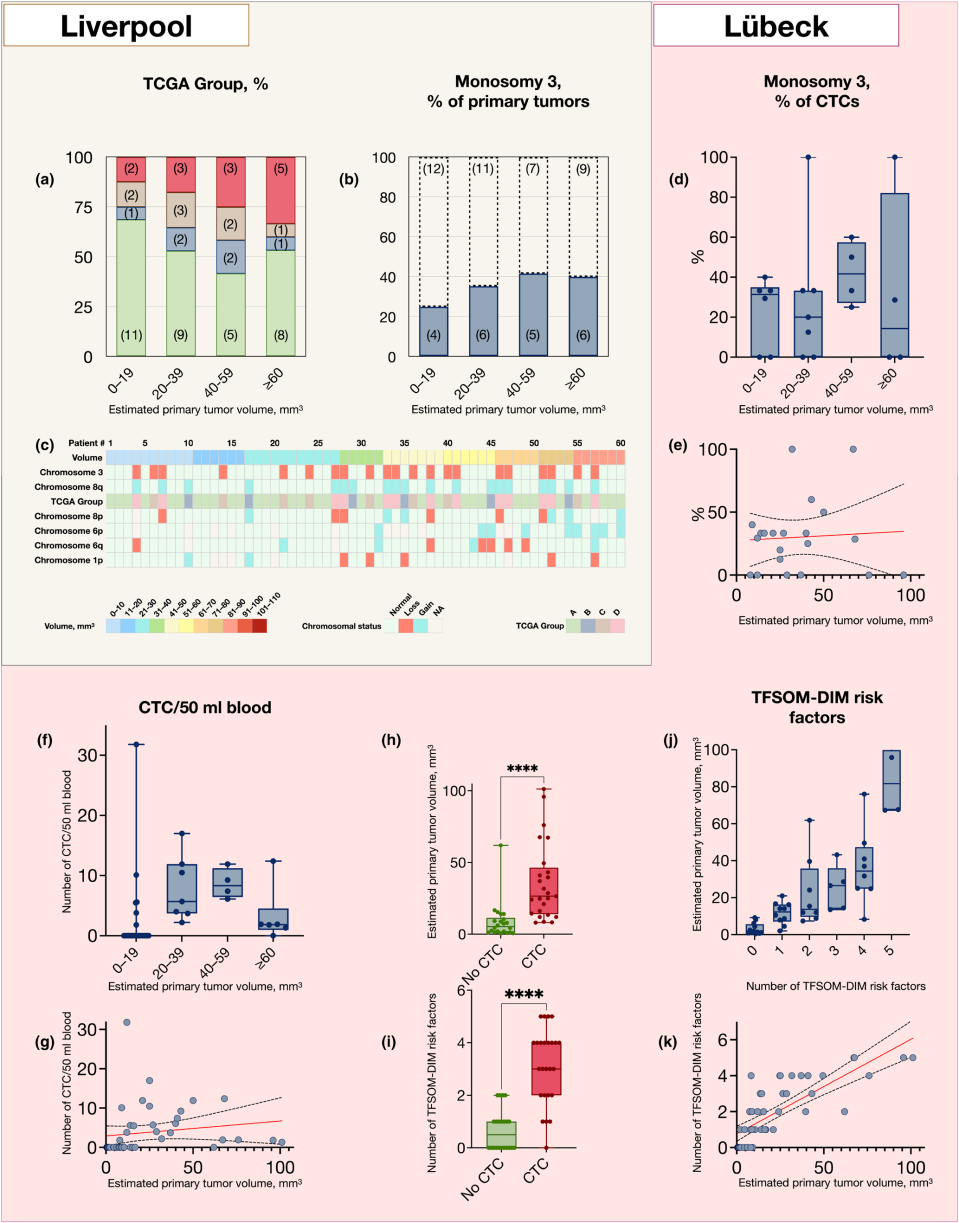
Tumour volume in relation to chromosomal aberrations, including The Cancer Genome Atlas (TCGA) group classification, and circulating tumour cells (CTC) in peripheral blood. (a) There was no significant trend in the distribution of TCGA groups between tumour size categories (Chi‐square test for trend, *p* = 0.59). The Y‐axis shows percentages; numbers in parentheses indicate the absolute number of tumours in each TCGA/volume category. (b) Similarly, there was no significant trend for an increasing proportion of monosomy 3 (Cochran‐Armitage test for trend, *p* = 0.44). (c) Chromosome 6p, 6q, and 8p aberrations occurred independently of chromosome 3 and 8q aberrations. Gain and loss of chromosome 1p were observed only in tumours with simultaneous monosomy 3 or gain of 8q. (d) In the Lübeck cohort, 22 of 44 patients (50%) had detectable CTC in peripheral blood. The number of CTC per 50 mL ranged from 1.3 to 31.8 (median 5.7). Of the 22 CTC‐positive patients, 15 had at least one CTC with monosomy 3, while the remaining 7 had only disomy 3 CTC. Within each tumour volume group, the mean percentage of CTC with monosomy 3 was 23% (SD, standard deviation, 18) for tumours ≤19 mm^3^, 28% (SD 43) for tumours 20–39 mm^3^, 42% (SD 16) for tumours 40–59 mm^3^, and 32% (SD 47) for tumours ≥60 mm^3^. (e) Linear regression showed no significant relationship between estimated primary tumour volume and the average proportion of CTC with monosomy 3 across patients in each group (*R* ^2^ = 0.004, *F* = 0.08, *p* = 0.79). In panels (d–e), chromosome 3 status refers to CTC; corresponding data from the primary tumour was not available in this cohort. (f, g) Similarly, there was no significant relationship between primary tumour volume and the number of CTC per 50 mL blood (*R* ^2^ = 0.03, *F* = 1.10, *p* = 0.30). (h) However, the estimated primary tumour volume was significantly greater in patients with detectable CTC, dichotomized as no CTC present versus any number of CTC present (Mann–Whitney *U*, *p* < 0.001). (i) The number of TFSOM‐DIM risk factors for the growth of choroidal nevi was also greater in those with detectable CTC (Mann–Whitney *U*; *p* < 0.001). (j, k) There was a significant linear relationship between primary tumour volume and the number of TFSOM‐DIM risk factors for the growth of choroidal nevi (Regression slope coefficient 0.05, *R* ^2^ = 0.62, *F* = 72.86, *p* < 0.001). CTC, circulating tumours cells; NA, not available; TCGA, The Cancer Genome Atlas group classification; TFSOM‐DIM, Thickness >2 mm on US, subretinal Fluid on optical coherence tomography (OCT), Symptoms of vision loss, Orange pigment on autofluorescence, Melanoma hollow on US, and DIaMeter >5 mm by photography. ****Mann–Whitney *U p* < 0.001.

Each tumour with available data on both chromosome 3 and 8q status in the Liverpool cohort (*n* = 60) was classified into one of four TCGA groups (A to D). Tumours were categorized by volume with cut‐offs selected to achieve a similar number of patients in each group: 13% of tumours ≤19 mm^3^, 18% of tumours 20–39 mm^3^, 25% of tumours 40–59 mm^3^, and 33% of tumours ≥60 mm^3^ fell into the D group, which has the poorest prognosis (Chi‐square test for trend for all size categories versus TCGA groups, *p* = 0.59). Additionally, 25% of tumours ≤19 mm^3^, 35% of tumours 20–39 mm^3^, 42% of tumours 40–59 mm^3^, and 40% of tumours ≥60 mm^3^ exhibited monosomy 3 (Cochran‐Armitage test for trend, *p* = 0.44). Chromosome 6p, 6q, and chromosome 8p aberrations occurred independently of chromosome 3 and 8q aberrations. Gain and loss of chromosome 1p were observed only in tumours with simultaneous monosomy 3 or gain of 8q (Figure 3a–c).

In the Lübeck cohort, 22 of 44 patients (50%) had detectable CTC in peripheral blood. CTC counts per 50 mL ranged from 1 to 32 (median, 6). Among CTC‐positive patients, the number of CTC with monosomy 3 ranged from 0 to 9, corresponding to 0%–100% of CTCs. Of the 22 CTC‐positive patients, 15 (68%) had >0% CTC with monosomy 3; the remaining 7 had only disomy 3. By tumour volume groups—≤19, 20–39, 40–59, and ≥60 mm^3^—the mean number of CTC with monosomy 3 per 50 mL was 3, 2, 4, and 1, respectively; the mean percentage of CTC with monosomy 3 (SD) was 23% (18), 28% (43), 42% (16), and 32% (47). Individual patient counts and proportions are provided in Table S2. These proportions should be interpreted cautiously given the small numbers within some volume strata and the low absolute CTC counts in several cases.

Linear regression analysis revealed no significant linear relationship between the estimated primary tumour volume and the percentage of CTC with monosomy 3 (*R* ^2^ = 0.004, *F* = 0.1, *p* = 0.79, Figure 3d,e). Similarly, there was no significant linear relationship between primary tumour volume and the number of CTC per 50 mL blood (*R* ^2^ = 0.03, *F* = 1.1, *p* = 0.30, Figure 3f,g). However, the estimated primary tumour volume was significantly greater in patients who had detectable CTC (dichotomized as no CTC present versus any number of CTC present, Mann–Whitney *U*, *p* < 0.001, Figure 3h). The number of TFSOM‐DIM risk factors for the growth of choroidal nevi was also greater in those with detectable CTC (Mann–Whitney *U*, *p* < 0.001, Figure 3i). There was a significant linear relationship between primary tumour volume and the number of TFSOM‐DIM risk factors for the growth of choroidal nevi. The slope was 0.05 per increasing mm^3^ (regression slope coefficient 0.05, 95% CI 0.04–0.06) with an intercept of 0.8 (95% CI 0.34–1.19, *R* ^2^ = 0.62, *F* = 72.9, *p* < 0.001, Figure 3j,k).

### 3.5 BAP1 expression, cytomorphology and risk factors for growth

**FIGURE 4:**
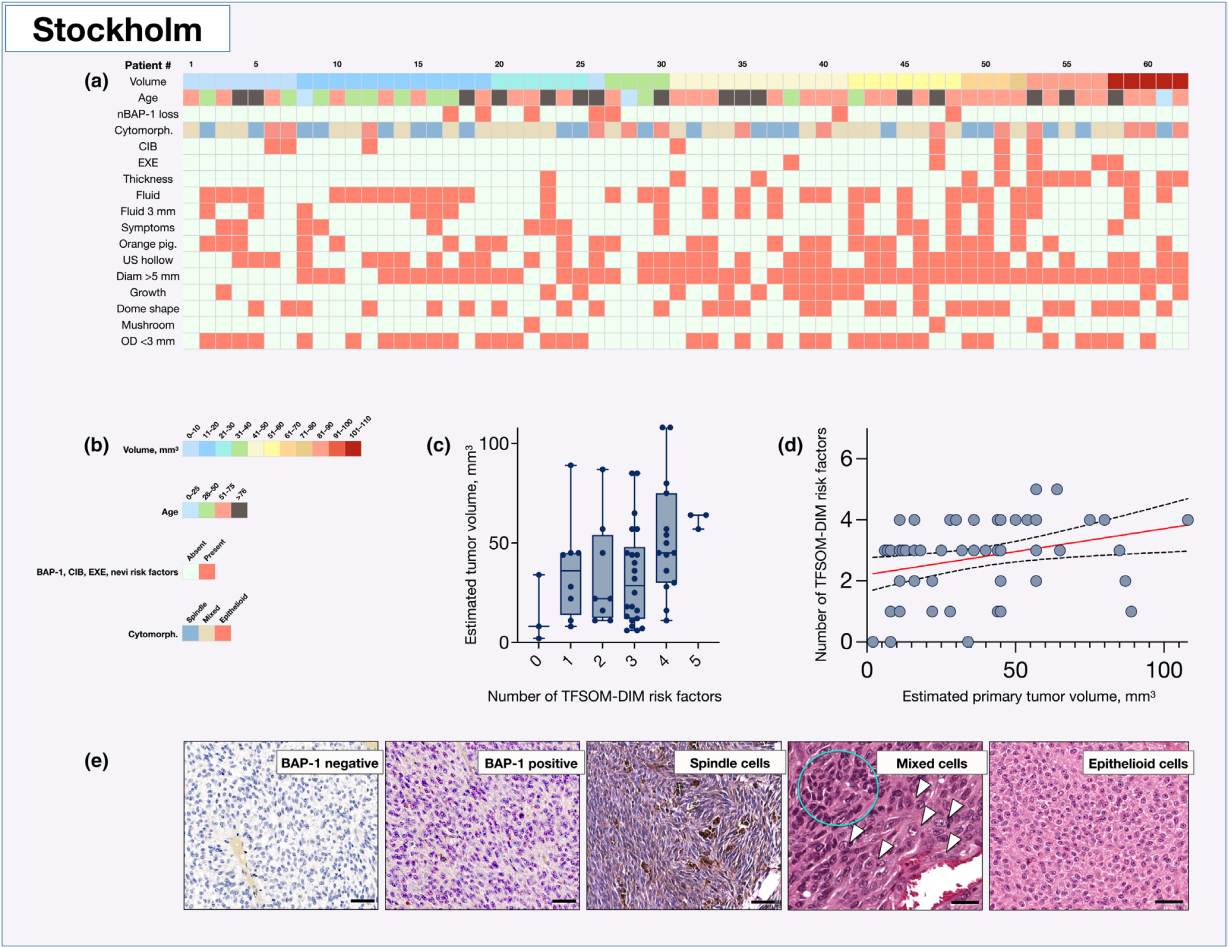
Clinicopathological characteristics and clinical risk factors for growth of choroidal nevi versus tumour volume. (a) Heat map illustrating the distribution of tumour volume (Volume), patient age at the time of uveal melanoma diagnosis (Age), loss of nuclear BAP1 expression (nBAP1 loss), cytomorphology (Cell), ciliary body involvement (CIB), extrascleral extension (EXE) and risk factors for growth of choroidal nevi. (b) Legend for the heatmap. (c, d) As for the Lübeck cohort, there was a significant linear relationship between primary tumour volume and the number of TFSOM‐DIM risk factors for the growth of choroidal nevi (regression slope coefficient 0.02, *R* ^2^ = 0.11, *F* = 6.7, *p =* 0.01). (e) Microphotographs showing immunohistochemical expression of BAP1 (BAP1 positive: Expression in ≥33% of tumour cell nuclei; BAP1 negative: Expression in <33% of tumour cell nuclei) and cytomorphology (spindle, mixed, and epithelioid). Scale bars represent 50 μm. CIB, ciliary body involvement; EXE, extrascleral extension; Fluid, subretinal fluid on OCT as cap over the lesion; Fluid 3 mm, subretinal fluid ≤3 mm from the lesion on OCT or autofluorescence (AF); Growth, lesion growth by at least 0.5 mm during observation; n/a, not available; Neg., negative/no; Orange pig., orange pigment visible on AF, in photos or upon biomicroscopy; Pos., positive/yes; Symptom, symptomatic vision loss ≤20/50 on Snellen visual acuity; Thickness, thickness >2 mm on ultrasonography; US hollow, ultrasound (US) hollowness. TFSOM‐DIM, Thickness >2 mm on US, subretinal Fluid on optical coherence tomography (OCT), Symptoms of vision loss, Orange pigment on autofluorescence, Melanoma hollow on US, and DIaMeter >5 mm by photography.

In the Stockholm cohort, TFSOM‐DIM clinical risk factors for the growth of choroidal nevi were collected alongside anatomic extent, immunohistochemical BAP1 expression, and cytomorphology for tumours that had undergone primary enucleation (*n* = 62). A correlation matrix was estimated to identify significant associations among these variables. After applying the Holm–Bonferroni correction for 78 comparisons, only one significant correlation was identified. Specifically, subretinal fluid within 3 mm of the lesion correlated with subretinal fluid observed as a cap over the lesion on OCT. These factors for the growth of choroidal nevi were evaluated even though the lesions were histopathologically confirmed as melanomas (Figure S3 and Table S3). Again, there was also a significant linear relationship between primary tumour volume and the number of TFSOM‐DIM risk factors (regression slope coefficient 0.02, 95% CI 0.003–0.03; intercept 2.2, 95% CI 1.7–2.8; *R* ^2^ = 0.11; *F* = 6.7, *p* = 0.01; Figure 4).

Next, we focused on orange pigment on AF and subretinal fluid on OCT—the two TFSOM‐DIM factors most strongly associated with lesion growth (Stalhammar, 2021). In the Stockholm cohort, neither orange pigment (univariate Fisher's exact tests *p* = 0.24, OR 4.0; 95% CI 0.6–45.1) nor subretinal fluid (*p* > 0.99, OR 1.2; 95% CI 0.2–8.9) was associated with loss of BAP1 expression. Similarly, in multivariable logistic regression including tumour volume, orange pigment nor subretinal fluid predicted loss of BAP1 expression (*p* > 0.29 for both).

In the Lübeck cohort, both orange pigment and subretinal fluid were strongly associated with CTC positivity on univariate analysis, and orange pigment alone was associated with monosomy 3–CTC before adjusting for tumour volume. CTC positivity occurred in 94% of eyes with orange pigment versus 32% without (*p* < 0.001) and in 88% with subretinal fluid versus 35% without (*p* = 0.002). Monosomy 3–CTC was present in 67% with orange pigment versus 24% without (*p* = 0.008) and in 60% with subretinal fluid versus 26% without (*p* = 0.026). In multivariable logistic regression including tumour volume, orange pigment on AF (adjusted OR = 18.1; 95% CI 2.2–403; *p* = 0.06) and subretinal fluid (OR = 7.1; 95% CI 1.0–65.8; *p* = 0.11) were not significant predictors of CTC positivity. For monosomy 3–CTC, orange pigment on AF had an adjusted OR = 5.4 (95% CI 1.2–27.9; *p* = 0.10), and subretinal fluid had an OR = 3.0 (95% CI 0.6–15.9; *p* = 0.37), adjusted for tumour volume.

## 4 DISCUSSION

### 4.1 Main findings

This study demonstrates that risk factors for aggressive behaviour in a significant proportion of small posterior UM. Several predictors, including monosomy 3, gain of 8q, loss of 6q, gain of 8p, and CTC appeared in tumours as small as 8 mm^3^. However, there was no significant relationship between commonly assessed clinical risk factors for the growth of choroidal nevi, such as subretinal fluid and orange pigment, and the markers for aggressive disease. This underlines the importance of tumour biopsies and genetic analyses, while highlighting the limitations of conventional clinical evaluations in predicting the prognostic severity of smaller tumours (Harbour et al., 2019).

In this context, an important question arises regarding how best to act on biopsy findings for small tumours, because the same results can prompt very different management decisions. One viewpoint suggests that if a biopsy reveals no aggressive features, there is little need for urgent intervention, whereas the detection of high‐risk markers (e.g. a *BAP1* mutation) warrants prompt treatment. Another perspective holds the opposite stance: that by the time aggressive traits are discovered, metastatic spread may already be inevitable, and that it is actually low‐risk lesions which should be treated without delay—before further mutations can emerge.

Previous research has reported a strong correlation between tumour volume and aggressive traits, including monosomy 3, gain of 8q, and epithelioid cytomorphology (Stalhammar et al., 2023). Similarly, Grisanti et al. found, based on a version of the Lübeck cohort used in this study, that among patients with CTC, a higher proportion of tumours with a diameter >5 mm and the presence of clinical risk factors for growth were presented (Grisanti et al., 2023). Our findings are consistent when dichotomising patients with small tumours into those with and without CTC. Thus, it is reasonable to interpret our results as indicating that across a wider range of tumour sizes there is a relationship between tumour volume and aggressive traits, but this association is attenuated when the analysis is restricted to small tumours. Moreover, clinical risk factors for nevus growth—such as orange pigment and subretinal fluid—were associated with CTC positivity and with the proportion of CTC harbouring monosomy 3 in univariable analyses, but these associations did not remain independent of tumour volume, and these clinical factors did not correlate with cytogenetic markers or loss of nuclear BAP1 expression.

Despite the presence of critical prognostic indicators like monosomy 3, gain of 8q, and loss of BAP1 expression in tumours smaller than 5 mm in LBD and less than 1.5 mm in thickness, other markers such as gain of 6p and loss of 1p were predominantly observed in slightly larger tumours. This pattern may suggest a clonal evolution within the tumour growth. As previously reported, loss of heterozygosity on chromosome 3 (LOH3) typically precedes mutations in *BAP1*, indicating a potential evolutionary pathway in tumorigenesis (Field et al., 2018). However, previous estimates of the sequence of cytogenetic events, such as monosomy 3 occurring early and losses of 1p, 8p, and gain of 8q as secondary events, are not clearly reflected in our findings from chromosomal testing of clinical specimens (Kilic et al., 2006). Early events like *BAP1* mutations and other canonical genomic aberrations have been interpreted to suggest that the metastatic proclivity of UM is set in stone early in tumour evolution (Field et al., 2018). While this interpretation is broadly accurate, our findings indicate that there are nuances. Aggressive traits may arise very early and are associated with an increased cumulative incidence of metastatic death. Nonetheless, as the tumour continues to grow, the prognosis becomes increasingly poor. Hussain and colleagues showed that patients with small (thickness ≤2.5 mm) monosomy 3 UM had a lower 5‐year absolute risk for metastatic mortality than those with larger (thickness >2.5 mm) monosomy 3 UM (Hussain et al., 2021). In two recent studies, we observed that longer diagnosis‐to‐treatment intervals in UM were linked to a higher cumulative incidence of metastatic death, and that larger primary tumours were linked to shorter overall survival following metastasis detection (Stalhammar, 2024; Yavuzyigitoglu et al., 2025). Therefore, although early tumour characteristics are crucial for prognosis, the patient's outcome is likely influenced by later events, tumour growth, and continued seeding of tumour cells into the circulation. It is reasonable to assume that these factors collectively modify prognosis.

Interestingly, while the gain of chromosome 6p has previously been associated with a favourable prognosis, our analysis of small tumours found no significant difference in the cumulative incidence of metastatic death between the 18 patients with 6p gains and the 50 patients with normal 6p. This lack of difference could be attributed to seven of these 18 patients also exhibiting monosomy 3, gain of 8q, or both, complicating the prognostic landscape. This observation raises important questions about the interplay of chromosomal changes, other aggressive traits, and their collective impact on the clinical outcome, particularly in the context of small melanomas.

### 4.2 Context

These findings are consistent with and add to several previous studies (Damato et al., 2010; Field et al., 2018; Jouhi et al., 2019; Uner et al., 2021). In the Small Fatal Choroidal Melanoma study, the smallest fatal tumour measured between 3.2 and 3.9 mm in diameter and 1 mm in thickness at the time of treatment (Jouhi et al., 2019). This would correspond, using the volume estimation method in our study, to approximately 5–7 mm^3^. This closely matches the smallest tumours with aggressive traits including monosomy 3, gain of 8q, loss of nuclear BAP1 expression, and CTC in the present study. This observation supports our previous estimation that *BAP1* mutations could potentially occur when the primary tumour is between just a few malignant cells to 6 mm^3^, based on observed intervals between mitoses (Uner et al., 2021).

It is well recognized that chromosomal abnormalities in UM can accumulate in diverse sequences (Damato et al., 2010; Field et al., 2018). For instance, loss of chromosome 3 might precede a gain in chromosome 8q and vice versa, with these changes potentially appearing at the tumour's inception or even within long‐standing melanomas that previously showed no detectable abnormalities. This variability in tumour progression means that lethal chromosomal abnormalities might develop at different stages across tumours.

Previous studies suggest that growth, defined as an increase in basal dimension of ≥0.3 mm or 0.5 mm in thickness, varies depending on the presence of certain risk factors. Growth is noted in 30% of lesions causing visual symptoms, 41% of lesions touching the optic disc, 36% of those thicker than 2 mm, 37% with subretinal fluid, and 34% with orange pigment, which, in some studies, has been associated with a higher risk for metastasis (Lorenzo et al., 2018; Shields et al., 2000). The presence of multiple risk factors increases the likelihood of growth, with 45%, 50%, 51% and 56% of lesions growing when 2, 3, 4, or all 5 of these factors are present, respectively (Shields et al., 2019). Multimodal imaging, particularly US and OCT, plays a crucial role in identifying these risk factors, with US effectively discerning low internal reflectivity and dome shape, and OCT excelling at evaluating subretinal fluid and detecting tumour extensions beyond what is detected in fundus photographs as well as tumours thinner than 1.5 mm (Shields et al., 2019).

However, it is important to note that not all observed growth indicates malignancy or metastatic potential. Mashayekhi et al. (2011) found that 31% of 284 choroidal nevi enlarged at a median rate of 0.06 mm per year, whereas Raval et al. (2021) reported that small choroidal melanomas grow at a rate of 1.8 mm per year. Jouhi et al. (2023) observed that the growth of melanocytic choroidal tumours, expressed as a percentage increase in LBD, was 1% in nevi and 34% in melanomas, further highlighting the significant difference in growth patterns between benign and malignant melanocytic lesions. It is essential to understand how these growth characteristics translate into long‐term outcomes. In 1979, Thomas et al. (1979) followed 65 patients with tumours ≤10 mm in diameter and ≤3 mm in thickness for 7.2 years, finding a 15‐year mortality rate of 14%. The smallest tumour linked to metastatic death measured 7 × 7 mm with a thickness of 2 mm at treatment, equivalent to a volume of 44 mm^3^ by our calculations. The study also reported a 15‐year mortality for spindle‐cell tumours at 5%, with no pure spindle A tumours identified.

Shields et al. (1995) reviewed 1329 patients with lesions ≤3 mm thick, noting metastases in 35 patients. Factors linked to metastases included posterior tumour margin touching the optic disc and documented growth, defined as an increase in basal dimension or thickness. The Collaborative Ocular Melanoma Study (COMS) (1997) noted an 8‐year melanoma mortality rate of 4% among 204 patients with choroidal melanomas measuring 1–3 mm thick and at least 5 mm in diameter. More recently, Malclès et al. (2015) found that 6 out of 59 tumours measuring ≤9 mm in diameter and ≤3 mm thick resulted in metastasis.

We believe that the small size of a lesion should not necessarily imply a passive approach. While smaller tumours are indeed less likely to exhibit aggressive genetic and cytogenetic features, early intervention may help prevent these aberrations from arising as they grow, as has been discussed extensively (Stalhammar, 2025; Stalhammar et al., 2024; Stalhammar et al., 2025; Witzenhausen & Stalhammar, 2024). Treating a higher proportion of tumours before they gain metastatic potential may reduce the cumulative incidence of metastatic death. However, this approach must be balanced against the risk of over‐treating benign lesions, hence exposing patients to unnecessary procedures and potential complications. The effective identification of clinical features distinguishing benign from potentially malignant lesions at an early stage is therefore essential.

### 4.3 Strengths and limitations

Our study benefits from a large cohort of 804 patients with small tumours from three major referral centres. In contrast to the hitherto largest study of small ciliochoroidal melanomas, this dataset includes patients who did not suffer metastatic deaths, enhancing our insights into potential thresholds for metastatic disease (Jouhi et al., 2019). The inclusion of detailed data on cytogenetic, genetic, cytomorphologic, and clinical risk factors, as well as CTC, has strengthened our analysis, providing a foundation for assessing prognosis and lesion growth. All tumours contributing TFSOM‐DIM data underwent histopathologic confirmation as melanoma, reducing verification bias and preventing differential misclassification that could arise if biopsy were performed more often in lesions with multiple risk factors.

We did not pool data from the three centres, which we consider advantageous because it allows examination of key factors in independent cohorts. Across cohorts, findings were concordant—for example, the relationship between tumour volume and the number of TFSOM‐DIM risk factors. Nonetheless, the retrospective, multicentre setting introduced heterogeneity in testing pipelines and data acquisition, and—critically—the availability of cytogenetic, immunohistochemical, and CTC data did not consistently overlap within individuals. As a result, denominators vary by analysis and several subgroup comparisons are based on small sample sizes. In addition, tumour *EIF1AX*, *SF3B1* and *BAP1* mutation data were unavailable, which may have nuanced some associations (including survival) and limited assessment of potential confounding by genotype.

A major limitation in this study is the small number of tumours measuring <3 mm in diameter and <1.5 mm in thickness included. Given that monosomy 3 was detected in a tumour as small as 3.3 mm in LBD and 1.4 mm in thickness—near the smallest size of the tumours analysed—it seems unlikely that a minimum size exists below which the absence of such aggressive features can be guaranteed. UM smaller than 3.3 mm are seldom diagnosed and are typically asymptomatic, reinforcing the challenge in establishing a definitive safe threshold for clinical application.

A smaller yet noteworthy limitation is that patients with iris melanoma were not included, even though previous research has shown that their prognosis is similar to that of posterior uveal melanoma when adjusted for both diameter and thickness (Sabazade, Gill, et al., 2021; Sabazade, Herrspiegel, et al., 2021).

Lastly, some may argue that our approach to avoiding type I errors—using both Holm–Bonferroni correction and adjusting the significance level by dividing 0.05 by the number of comparisons per cohort—is overly conservative and may increase the risk of type II errors. For instance, without these adjustments, the competing risk incidence of metastatic death associated with the loss of chromosome 1p, loss or gain of 8p, and gain of 8q would have reached statistical significance. Given the number of comparisons made in this study and the potential correlations between outcomes (e.g. an indicator of metastatic death used in survival analysis and a measure of tumour size in linear regression), we deemed it prudent to both adjust *p* values upwards and significance levels downwards.

## 5 CONCLUSIONS

Cytogenetic aberrations, loss of BAP1 expression, and circulating tumour cells are present in a substantial proportion of small uveal melanomas, and several aggressive traits occur in tumours with volumes <10 mm^3^. In the genotyped subset, monosomy 3 was observed in 23% (41/175), 8q gain in 32% (23/72) and 6q aberrations in 13% (9/71); loss of nuclear BAP1 was seen in 22% (19/88) of tumours assessed; and CTC were detected in 50% (22/44). Within the tested range (0–108 mm^3^), we found no size threshold below which aggressive traits were absent. Moreover, monosomy 3, 6q aberrations, and loss of BAP1 were each associated with a higher cumulative incidence of metastatic death. By contrast, clinical risk factors traditionally used to predict nevus growth—such as orange pigment and subretinal fluid—appear to have a stronger association with lesion size than with cytogenetic or BAP1‐based markers of metastatic potential. These findings support the need for improved clinical markers and minimally invasive assays to identify metastatic risk in small tumours.

## FUNDING INFORMATION

Support for this study was provided to Gustav Stålhammar from: Region Stockholm (RS‐2019‐1138) and The Swedish Cancer Society (232 613 Fk). The sponsors or funding organizations had no role in the design or conduct of this study research.

## CONFLICT OF INTEREST STATEMENT

Gustav Stålhammar: None, Helen Kalirai: None, Sarah E Coupland: None, Salvatore Grisanti: None, Svenja Rebecca Sonntag: None, Ayseguel Tura: None, Antonio Eleuteri: None, Rumana N. Hussain: None, Heinrich Heimann: None, Hannah Coudé Adam: None.

## Supporting information


Data S1.

Figure S1.
