# Supplementary material for: Metastatic potential of small posterior uveal melanomas
Source: Acta Ophthalmol. 2025 Aug 21;104(2):e191–203. doi: 10.1111/aos.17582 (PMC12888941; doi:10.1111/aos.17582)
Supplement: Supplementary file 2 — Figure S1. [file AOS-104-e191-s001.pdf]

Supplementary material

Metastatic Potential of Small Posterior Uveal Melanomas

Gustav Stålhammar MD, PhD[1,2,3], Helen Kalirai BSc, PhD [4,5], Sarah E Coupland MBBS, PhD[4,5], Salvatore Grisanti MD, PhD[6], Svenja Rebecca Sonntag MD, PhD[6], Ayseguel Tura MSc, PhD[6], Antonio Eleuteri PhD[7], Rumana N Hussain MBBS, MD[4,8] , Heinrich Heimann MD, PhD[4,8], Hannah Coudé Adam MD, PhD[1]

[1]Department of Clinical Neuroscience, Division of Eye and Vision, Karolinska Institutet, Stockholm, Sweden
[2]Ocular Oncology Service, St. Erik Eye Hospital, Stockholm, Sweden
[3]St. Erik Ophthalmic Pathology Laboratory, St. Erik Eye Hospital, Stockholm, Sweden
[4]Liverpool Ocular Oncology Research Group, Department of Eye and Vision Science, University of Liverpool, Liverpool, United Kingdom
[5]Liverpool Clinical Laboratories, NHS University Hospitals of Liverpool Group, United Kingdom
[6]Department of Ophthalmology, University Medical Center Schleswig-Holstein, Luebeck, Germany
[7]Research & Innovation Department, NHS University Hospitals of Liverpool Group, Liverpool, United Kingdom
[8]Liverpool Ocular Oncology Centre, NHS University Hospitals of Liverpool Group, Liverpool, United Kingdom

Table of contents

SUPPLEMENTARY FIGURE 1 2
SUPPLEMENTARY TABLE 1A 3
SUPPLEMENTARY TABLE 1B 4
SUPPLEMENTARY FIGURE 2 5
SUPPLEMENTARY TABLE 2 6
SUPPLEMENTARY FIGURE 3 7
SUPPLEMENTARY TABLE 3 8

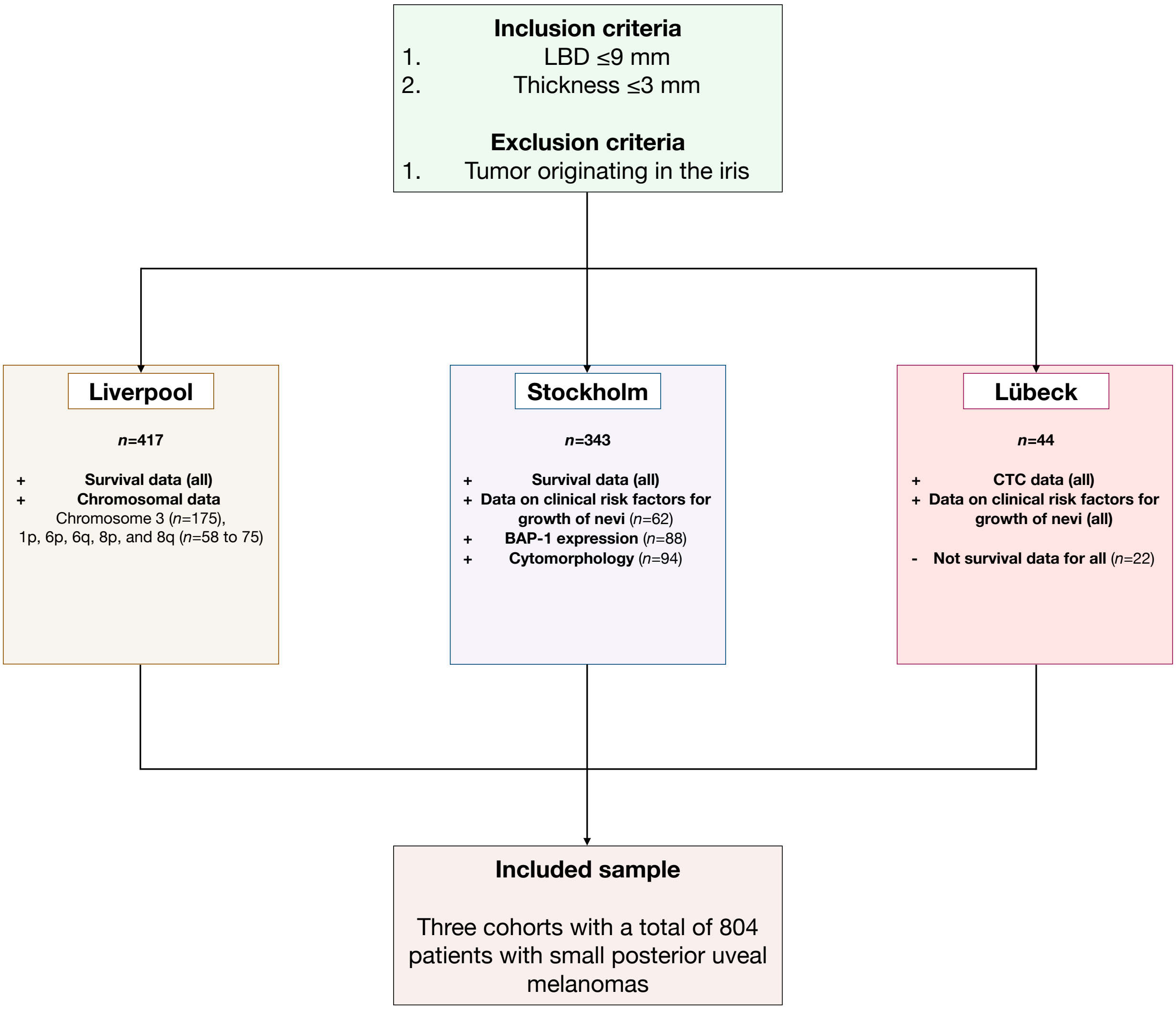

Supplementary Figure 1
Flow chart of included patients and tumors, summarizing the data available from each cohort. Abbreviations: CTC, circulating tumor cells; LBD, largest basal tumor diameter.

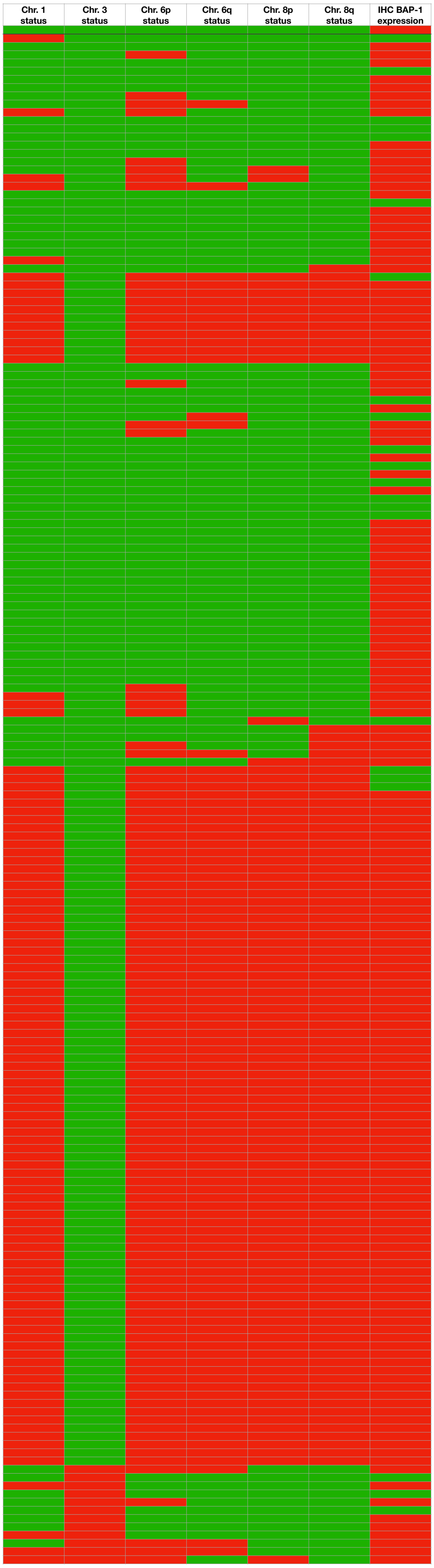

Supplementary Table 1A
Available (green) and unavailable (red) chromosomal data and immunohistochemical BAP-1 expression in the Liverpool cohort. Each row represents one patient. All 417 patients had data on tumor size (largest basal diameter and thickness), presence or absence of ciliary body involvement and extraocular extension, length of follow-up, and vital status at last follow-up. Chromosomal status and/or BAP-1 expression (at least one parameter) were available only for the 187 patients shown; no other patients had these data. Chr., chromosome.

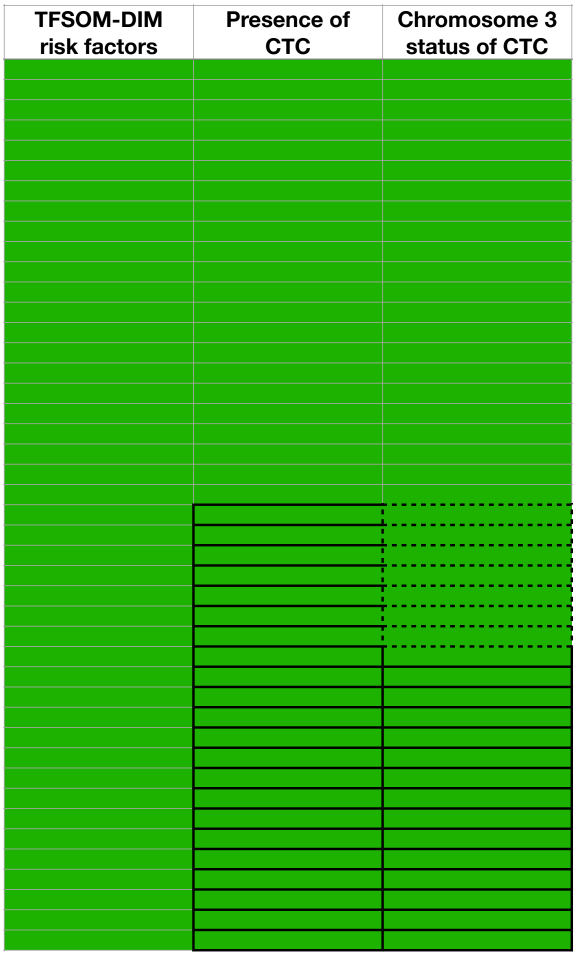

Supplementary Table 1B
Available (green) and unavailable (red) data in the Lübeck cohort. All patients had available data on which TFSOM-DIM risk factors were present or absent. These include: Thickness >2 mm on ultrasound, subretinal Fluid on optical coherence tomography (OCT), visual Symptoms, Orange pigment on autofluorescence, Melanoma hollow on ultrasound, and largest basal DIAmeter >5 mm on photography. Data were also available on the presence or absence of circulating tumor cells (CTCs), and—if CTCs were detected—on their chromosome 3 status (normal/euploidy or loss). Cells with a black border in the “Presence of CTC” column indicate patients who were CTC-positive. Cells without a black border indicate no detected CTCs. In the “Chromosome 3 status of CTC” column, a black border marks monosomy 3, while a dashed border indicates disomy 3. Chromosome 3 status was not available for primary tumors or metastases.

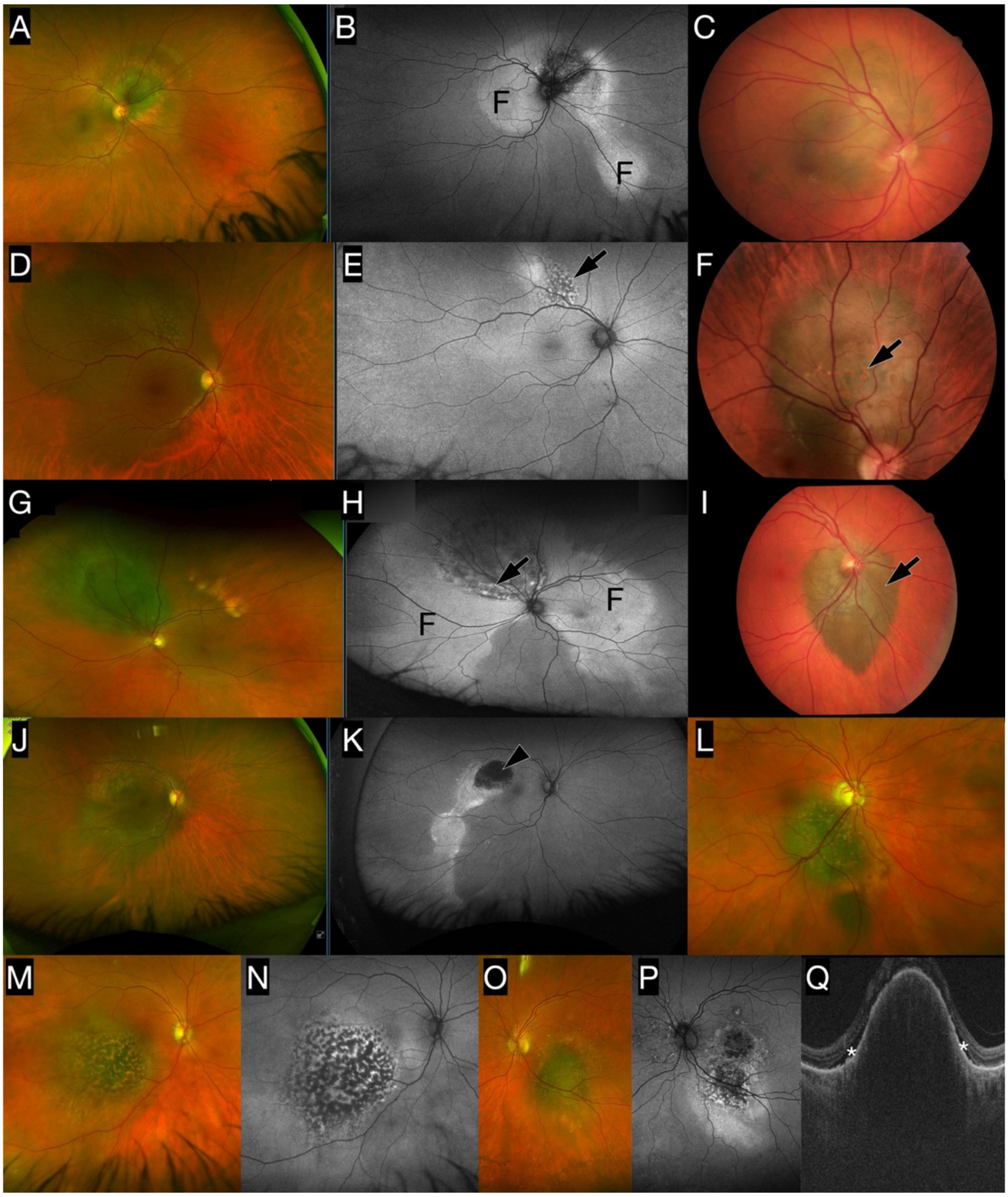

Supplementary Figure 2

Examples of small choroidal melanomas and clinical risk factors. A) A juxtapapillary tumor with a depigmented halo. B) In autofluorescence, subretinal fluid (F) can be seen with macular and inferotemporal extension. C) Another juxtapapillary tumor, partially engulfing the optic disc. D) A large but flat tumor with extension under the macula and superiotemporally. E) In autofluorescence, hyperautofluorescent specks of orange pigment (arrow) are clearly visible in one part of the tumor. F) Another juxtapapillary tumor with orange pigment that is clearly visible in color photo (arrow). G) A juxtapapillary tumor with H) orange pigment (arrow) and extensive fluid (F) visible on autofluorescence. I) A circumpapillary tumor with some hints of orange pigment in its nasal portion (arrow). J) A tumor in which the central is seen with K) hypoautofluorescence from retinal pigment epithelium (RPE) atrophy (arrowhead). L) A tumor with orange pigment and seemingly a small inferior satellite lesion. M and N) A tumor with orange pigment mimicking leopard spots. O and P) A tumor under the macula with orange pigment and subretinal fluid. Q) The presence of subretinal fluid was confirmed with optical coherence tomography (OCT, asterisks). (A,B), (D,E), (G,H), (J,K), (M, N), and (O, P, Q) depict the same tumor.

CTC per 50 ml blood, *n*	CTC with Monosomy 3 per 50 ml blood, *n*	Classification	CTC with Monosomy 3 (%)
1.8	0.0	Disomy 3	0
10.1	4.0	Monosomy 3	40
3.8	0.0	Disomy 3	0
31.8	9.3	Monosomy 3	29.4
5.6	1.9	Monosomy 3	33.3
5.5	1.8	Monosomy 3	33.3
11.9	4.0	Monosomy 3	33.3
17	2.1	Monosomy 3	12.5
10.5	2.1	Monosomy 3	20
5.7	1.9	Monosomy 3	33.3
4	0.0	Disomy 3	0
2.2	2.2	Monosomy 3	100
3.7	0.0	Disomy 3	0
6.1	2.0	Monosomy 3	33.3
7.4	1.9	Monosomy 3	25
9.25	5.6	Monosomy 3	60
11.9	6.0	Monosomy 3	50
1.9	1.9	Monosomy 3	100
12.4	3.5	Monosomy 3	28.6
1.9	0.0	Disomy 3	0
1.8	0.0	Disomy 3	0
1.3	NA		NA

Supplementary Table 2
Number of circulating tumor cells (CTC) and proportion of CTC with monosomy 3 for the 22 patients with positive CTC in the Lübeck cohort. The remaining 22 patients had no detectable CTC. NA, not available.

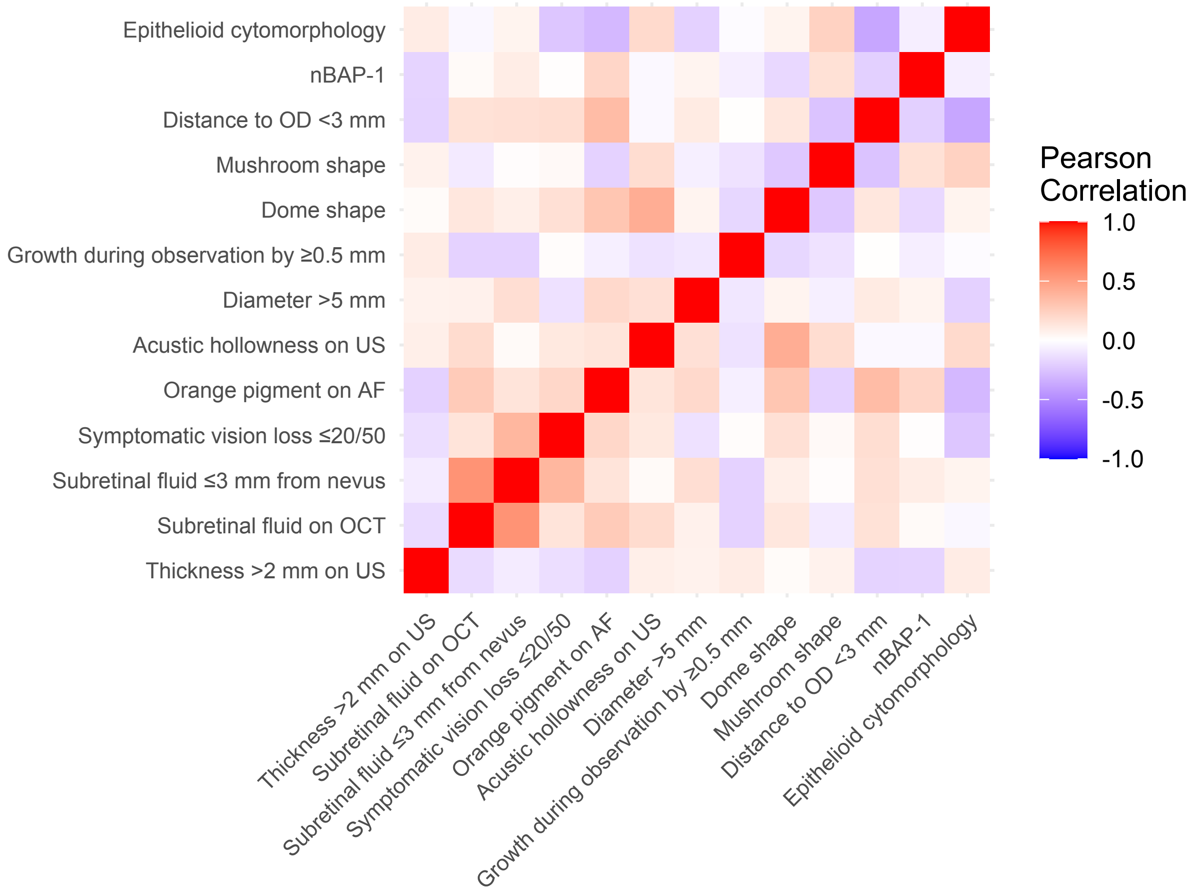

Supplementary Figure 3
Heatmap of correlation coefficients between clinical risk factors for growth of choroidal nevi, BAP-1 status, and cytomorphology. Each cell shows the correlation for the relationship between the variables on the x and y axes. Color intensity indicates the strength of the correlation, with warmer colors indicating stronger positive correlations and cooler colors indicating stronger negative correlations. AF, autofluorescence. OCT, optical coherence tomography. nBAP-1, loss of BAP-1 expression in tumor cell nuclei. OD, optic disc. US, ultrasonography.

Factor	Thickness >2 mm on US	Subretinal fluid on OCT	Subretinal fluid ≤3 mm from nevus	Symptomatic vision loss ≤20/50	Orange pigment on AF	Acoustic hollowness on US	Diameter >5 mm	Growth during observation at least 0.5 mm	Dome shape	Mushroom shape	Distance to OD <3 mm	BAP-1 loss
Thickness >2 mm on US												
Subretinal fluid on OCT	-0.15, *P*>0.99											
Subretinal fluid ≤3 mm from nevus	-0.08, *P*>0.99	**0.55, *P*<0.001**										
Symptomatic vision loss ≤20/50	-0.14, *P*>0.99	0.14, *P*>0.99	0.38, *P*=0.20									
Orange pigment on AF	-0.20, *P*>0.99	0.27, *P*>0.99	0.14, *P*>0.99	0.21, *P*>0.99								
Acustic hollowness on US	0.08, *P*>0.99	0.18, *P*>0.99	0.03, *P*>0.99	0.12, *P*>0.99	0.14, *P*>0.99							
Diameter >5 mm	0.07, *P*>0.99	0.07, *P*>0.99	0.17, *P*>0.99	-0.13, *P*>0.99	0.20, *P*>0.99	0.16, *P*>0.99						
Growth during observation at least 0.5 mm	0.10, *P*>0.99	-0.19, *P*>0.99	-0.19, *P*>0.99	0.01, *P*>0.99	-0.07, *P*>0.99	-0.13, *P*>0.99	-0.10, *P*>0.99					
Dome shape	0.02, *P*>0.99	0.13, *P*>0.99	0.08, *P*>0.99	0.16, *P*>0.99	0.30, *P*>0.99	0.42, *P*=0.05	0.06, *P*>0.99	-0.17, *P*>0.99				
Mushroom shape	0.07, *P*>0.99	-0.09, *P*>0.99	0.01, *P*>0.99	0.03, *P*>0.99	-0.19, *P*>0.99	0.18, *P*>0.99	-0.07, *P*>0.99	-0.12, *P*>0.99	-0.23, *P*>0.99			
Distance to OD <3 mm	-0.19, *P*>0.99	0.15, *P*>0.99	0.16, *P*=0.38	0.18, *P*>0.99	0.35, *P*>0.99	-0.03, *P*>0.99	0.11, *P*>0.99	0.01, *P*>0.99	0.13, *P*>0.99	-0.26, *P*>0.99		
BAP-1	-0.18, *P*>0.99	0.03, *P*>0.99	0.09, *P*>0.99	0.01, *P*>0.99	0.21, *P*>0.99	-0.03, *P*>0.99	0.06, *P*>0.99	-0.07, *P*>0.99	-0.16, *P*>0.99	0.16, *P*>0.99	-0.20, *P*>0.99	
Epithelioid cytomorphology	0.10, *P*>0.99	-0.03, *P*>0.99	0.06, *P*>0.99	-0.25, *P*>0.99	-0.30, *P*>0.99	0.19, *P*>0.99	-0.20, *P*>0.99	-0.01, *P*>0.99	0.06, *P*>0.99	0.24, *P*>0.99	-0.38, *P*=0.16	-0.07, *P*>0.99

Supplementary Table 3

Pearson correlation coefficients and corresponding *P* values for the correlation matrix shown in Supplementary Figure 3. Significant correlations are indicated in bold. All *P* values have been corrected using the Holm-Bonferroni method. AF, autofluorescence; OCT, optical coherence tomography; OD, optic disc; US, ultrasonography.
